# Supplementary material for: Inconspicuous breeding coloration to conceal eggs during mouthbrooding in male cardinalfish
Source: iScience. 2024 Dec 12;27(12):111490. doi: 10.1016/j.isci.2024.111490 (PMC11700633; doi:10.1016/j.isci.2024.111490)
Supplement: Document S1. Figures S1–S11 and Table S3 [file mmc1.pdf]

iScience, Volume *27*

Supplemental information

Inconspicuous breeding coloration to conceal eggs during mouthbrooding in male cardinalfish

Hikaru Ishihara and Shinji Kanda

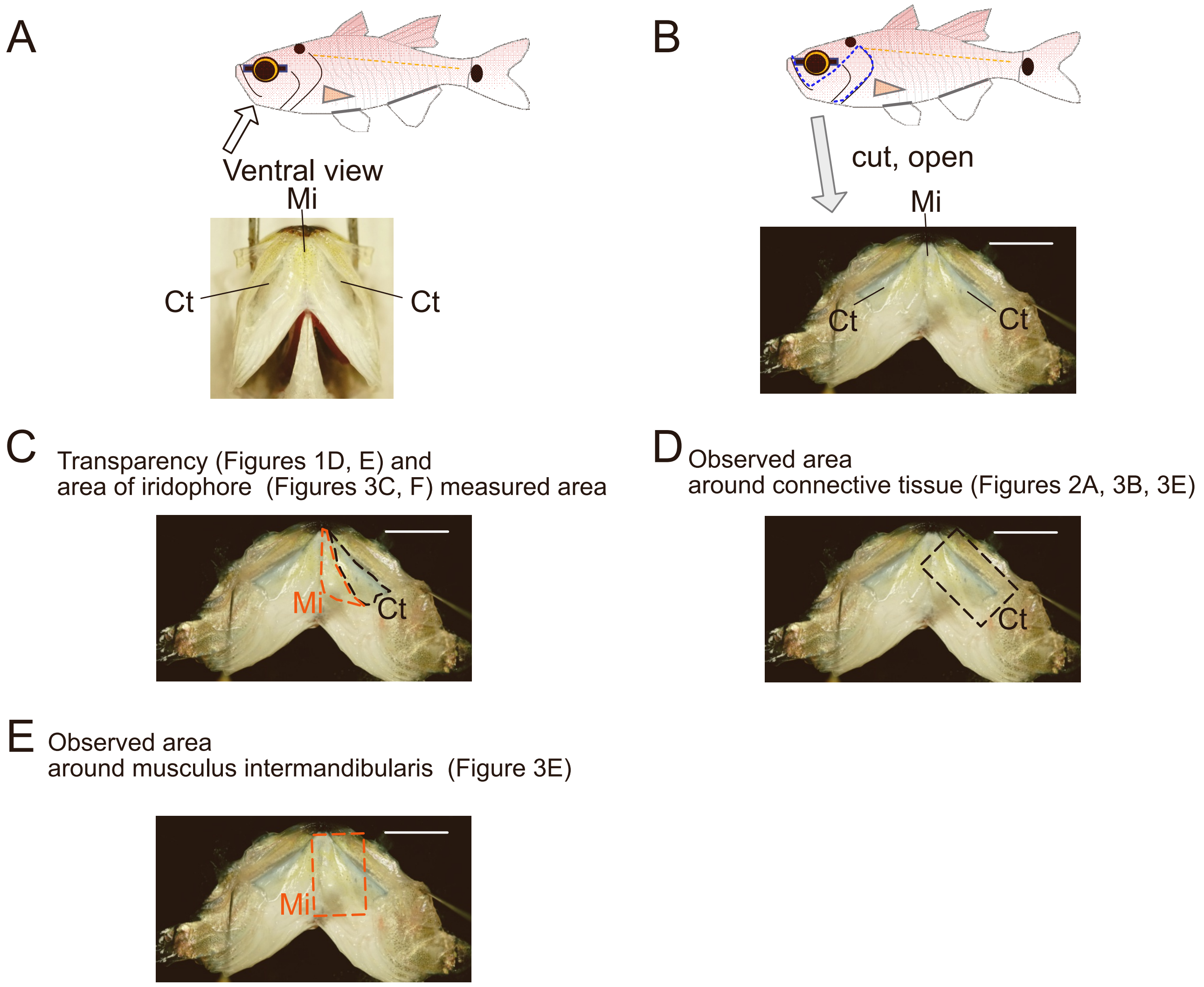

Figure S1 Description of the lower jaw region used in each observation and analysis, related to Figures 1, 2, and 3.

(A) Ventral view of the lower jaw with the mouth open. The photograph was taken in the area marked with white arrows. (B) The isolated lower jaw cut out along the blue dashed line on both sides of the body. After the incision was made, the lower jaw was opened so that the outside of the cavity was up. (C) Transparency (Figures 1D and 1E) and area of the iridophore (Figures 3C and 3F) measured region. The black dashed line indicates the region measured as Ct. The orange dashed line indicates the region measured as Mi. (D) The black dashed line indicates the observed region around Ct (Figures 2A, 3B, and 3E). (E) The orange dashed line indicates the observed region around Mi (Figure 3E). Ct, the connective tissue in the lower jaw; Mi, the musculus intermandibularis in the lower jaw. Scale bar, 10 mm.

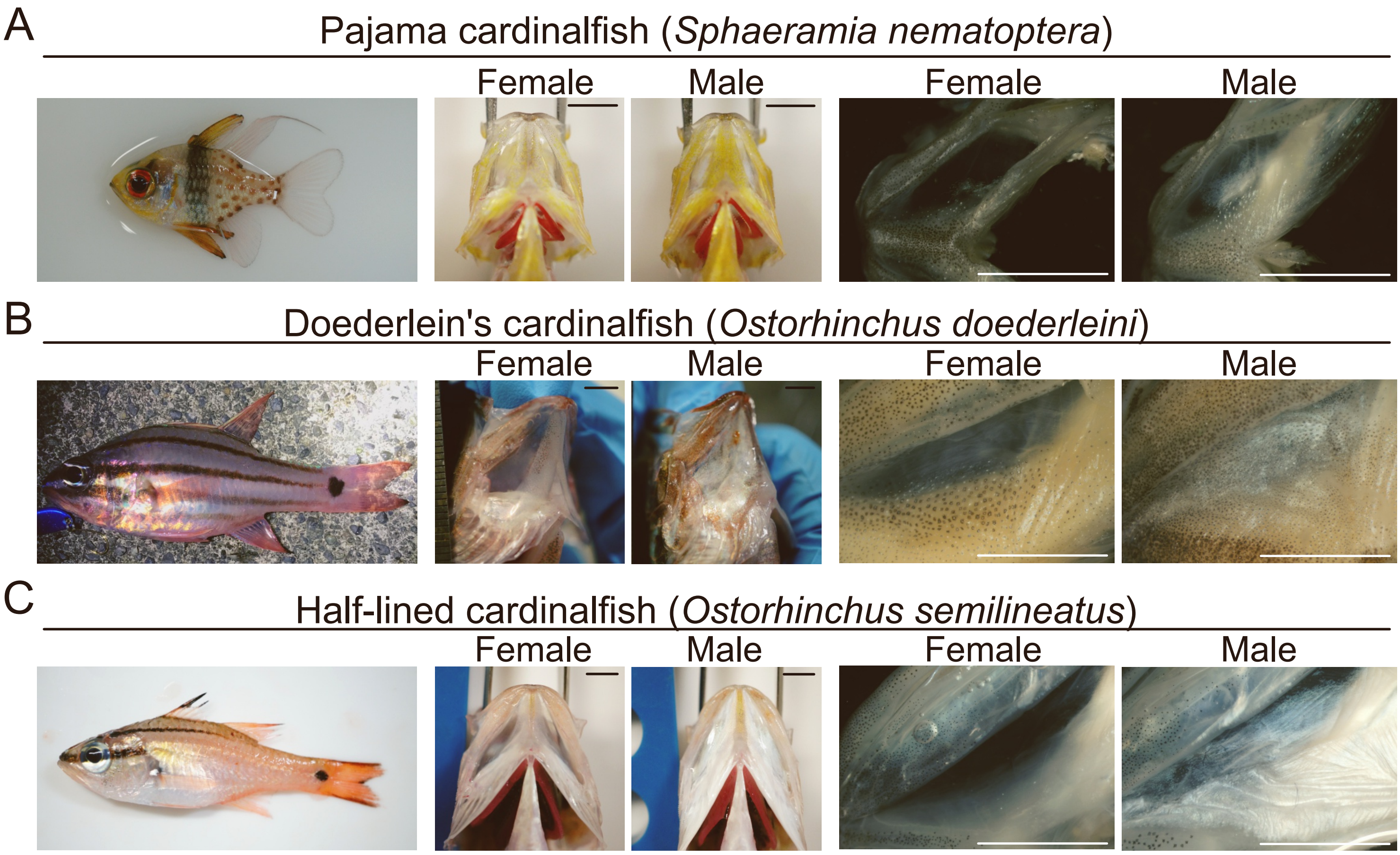

Figure S2 The lower jaws of other male Apogoninae fishes also contain iridophores, related to Figure 1.

(A) Pajama cardinalfish (*Sphaeramia nematoptera*), (B) Doederlein's cardinalfish (*Ostorhinchus doederleini*), and (C) Half-lined cardinalfish (*Ostorhinchus semilineatus*).

The left column shows representative whole-body photographs of each fish. Note that the right photographs show enlarged views of the same individuals as middle photographs after fixation with 4% PFA. Scale bar, 5 mm.

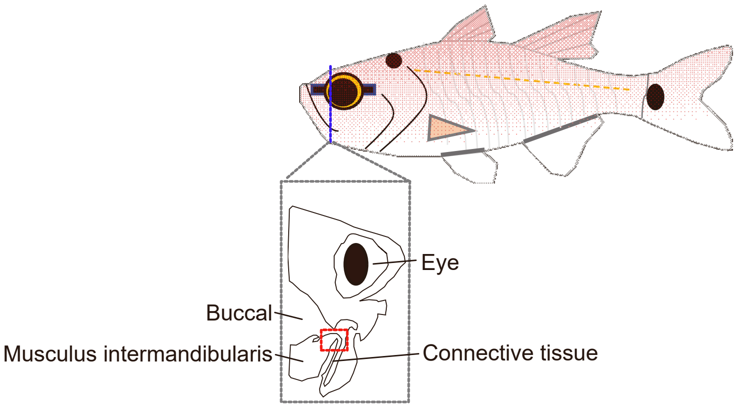

Figure S3 Schematic illustration of the sectioning site, related to Figures 2 and 4.

The frontal illustration surrounded by a gray dashed line indicates the frontal plane of the blue dashed line on the fish illustration. The area surrounded by the red dashed line indicates the area observed in Figures 2B and 4G.

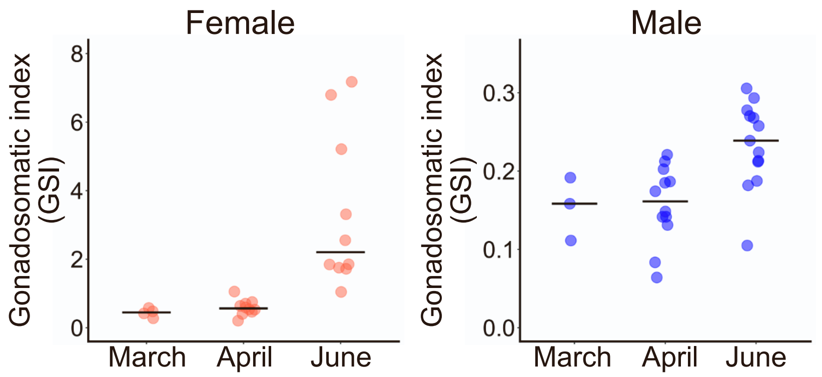

Figure S4 Seasonal changes in the gonadosomatic index (GSI) of males and females, related to Figure 3.

Gonadal size increases as the breeding season approaches. Bars indicate the medians. females in March: n = 4 males in March: n = 3; females in April: n = 10; males in April: n = 12; females in June: n = 10; males in June: n = 13. Due to the small number of samples in March, statistical tests have not been performed.

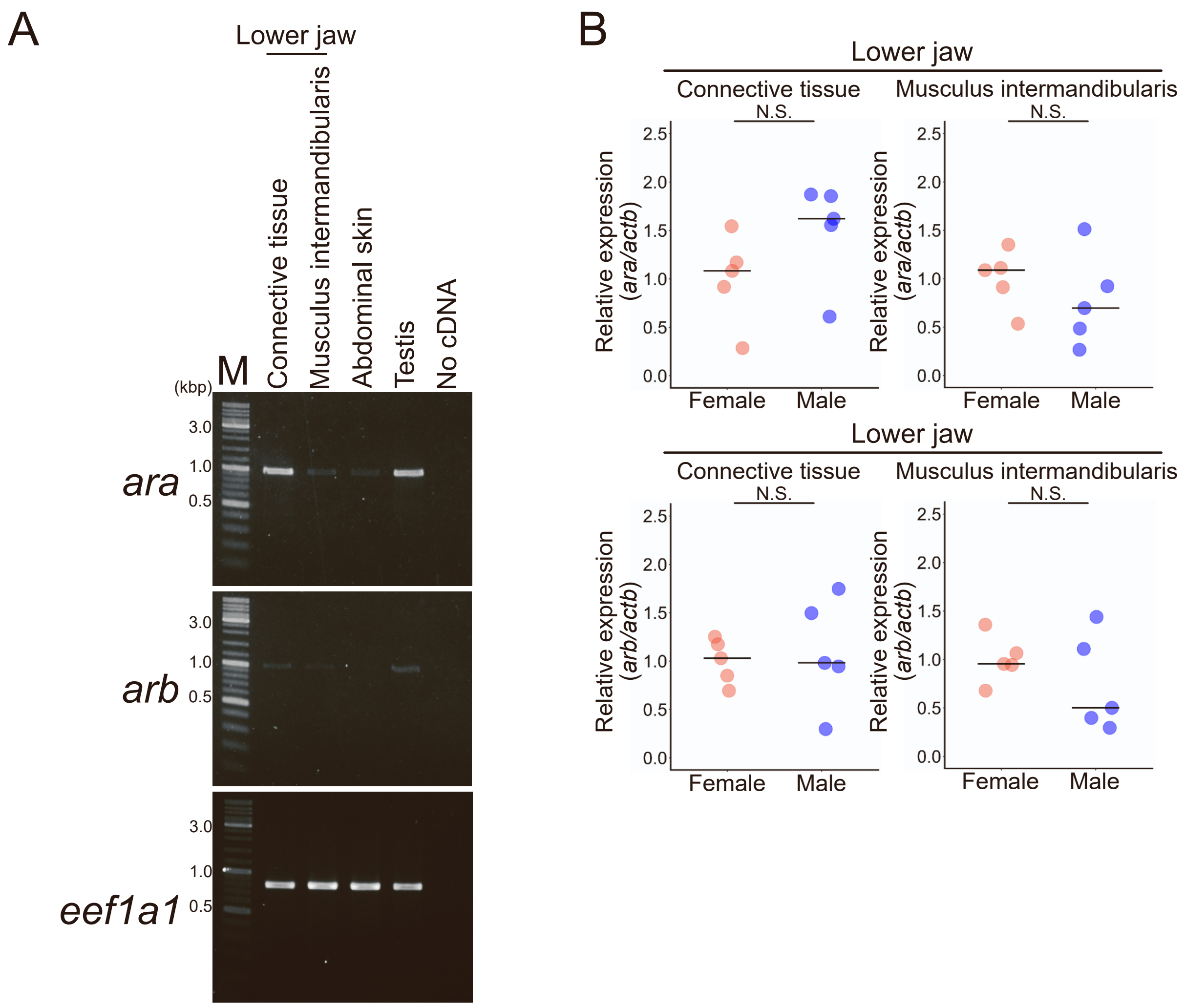

Figure S5 The genes encoding androgen receptors are expressed in both male and female lower jaws, related to Figure 3.

(A) RT–PCR for *androgen receptor a* (*ara*) and *b* (*arb*) in male fish. The expression of *ara* was detected in the connective tissue and the musculus intermandibularis in the lower jaw. A small amount of the expression of *arb* was detected in the connective tissue and the musculus intermandibularis in the lower jaw. (B) The expression levels of *ara* and *arb* in the connective tissue and the musculus intermandibularis in the lower jaw were not significantly different between females and males. Bars indicate the medians. N.S., not significant, Wilcoxon rank sum test; n = 5.

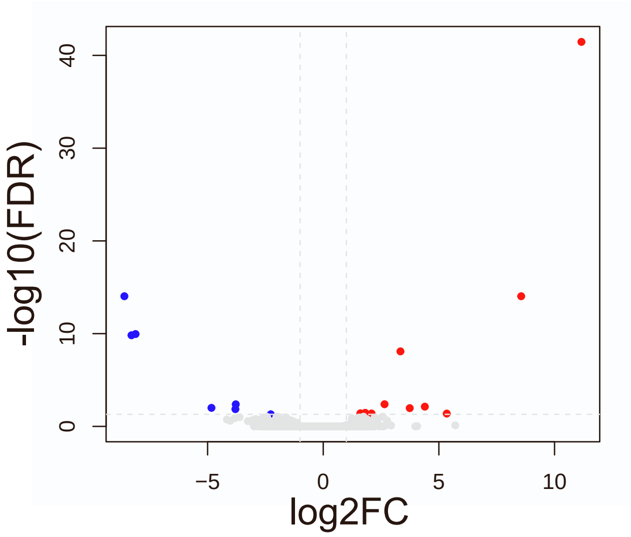

Figure S6 Volcano plot of differentially expressed genes (DEGs) from RNA-seq analysis of the musculus intermandibularis in the lower jaw between EtOH-treated females and 33 nM methyl testosterone-treated females on day 3, related to Figure 4.

Positive log2 fold-change (FC) values correspond to increased expression in females treated with 33 nM methyl testosterone. The vertical lines indicate a log2FC threshold of 1, while the horizontal line indicates a -log10 false discovery rate (FDR) threshold of -log10(0.05).

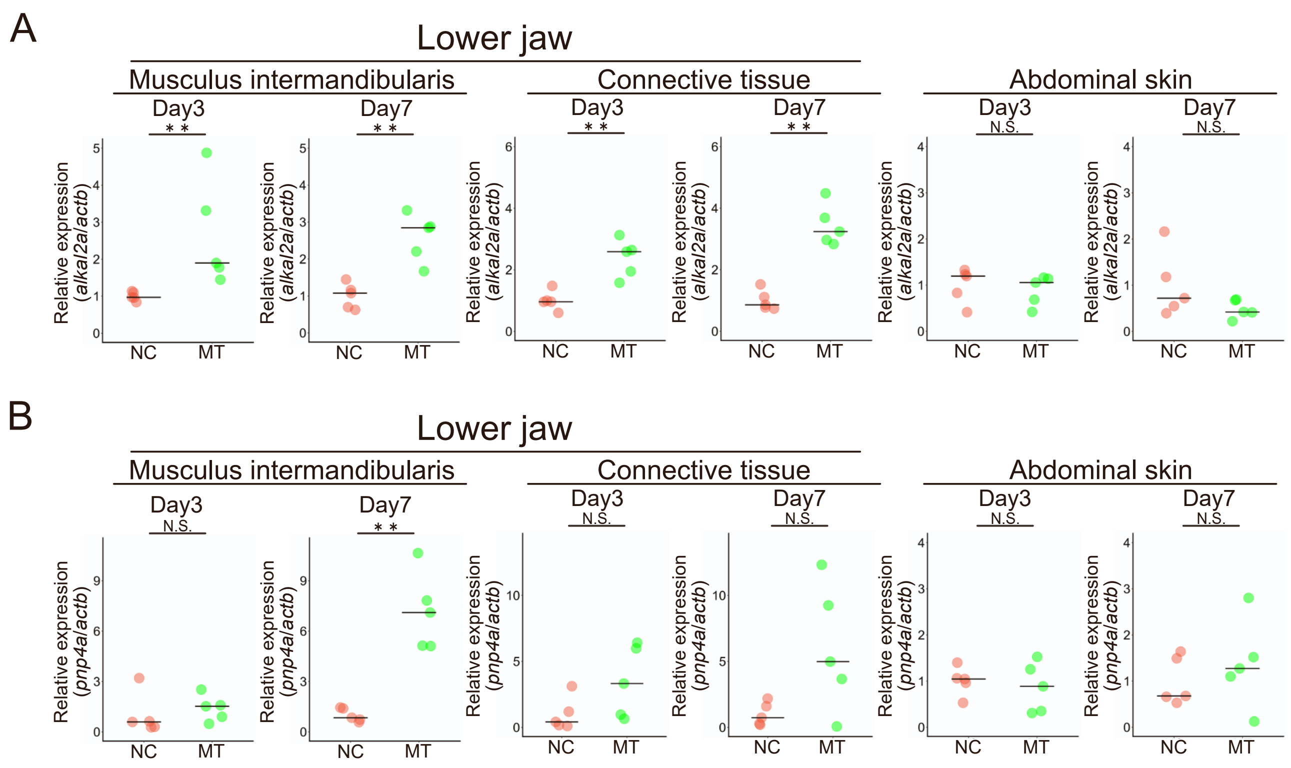

Figure S7 Expression of *alkal2a* is upregulated by androgen prior to iridophore development, specifically in the lower jaw, related to Figure 4.

(A) The expression level of *alk and ltk ligand 2a* (*alkal2a*) of 33 nM methyl testosterone-treated females (MT) on days 3 and 7 is significantly greater than that of EtOH-treated females (NC) in the musculus intermandibularis and the connective tissue in the lower jaw, whereas it is not significantly different in other tissues, such as the abdominal skin. Bars indicate the medians. **, $p < 0.01$, N.S., not significant, Wilcoxon rank sum test; $n = 5$. (B) The expression level of *purine nucleoside phosphorylase 4a* (*pnp4a*) of 33 nM methyl testosterone-treated females (MT) on day 7 is significantly greater than that of EtOH-treated females (NC) in the musculus intermandibularis in the lower jaw, whereas it was not significantly different on day 3. The expression levels of *pnp4a* in the connective tissue in the lower jaw and abdominal skin does not differ between 33 nM methyl testosterone-treated females (MT) and EtOH-treated females (NC) on days 3 and 7. Bars indicate the medians. **, $p < 0.01$, N.S., not significant, Wilcoxon rank sum test; $n = 5$.

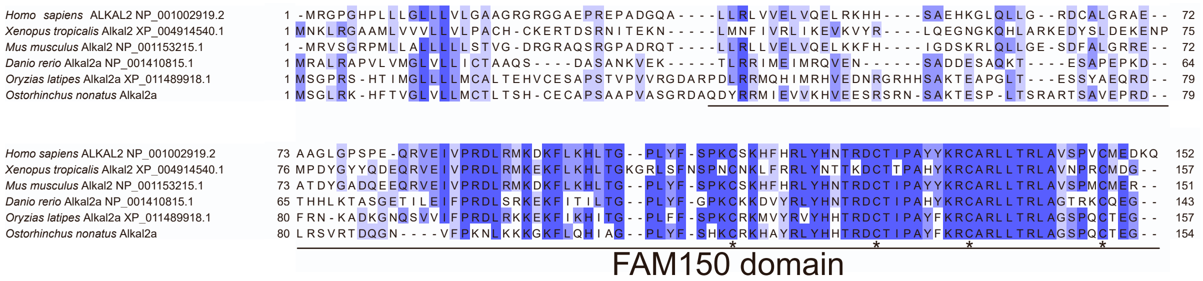

Figure S8 Alignment of Alkal2 proteins, related to Figure 4.

The underline indicates the FAM150 domain predicted in Alkal2a of spotnape cardinalfish. Asterisks indicate four conserved cysteines essential for maintaining structural integrity.

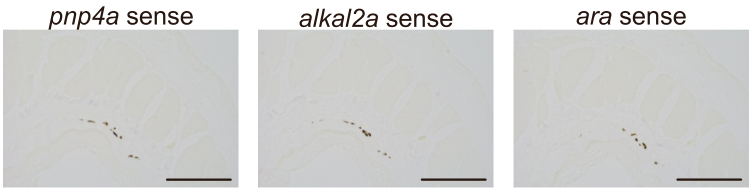

Figure S9 Confirmation of the specificity of mRNA probes using sense probes, related to Figure 4.

No purple signals are detected with the control sense probe. Scale bar, 100 μm.

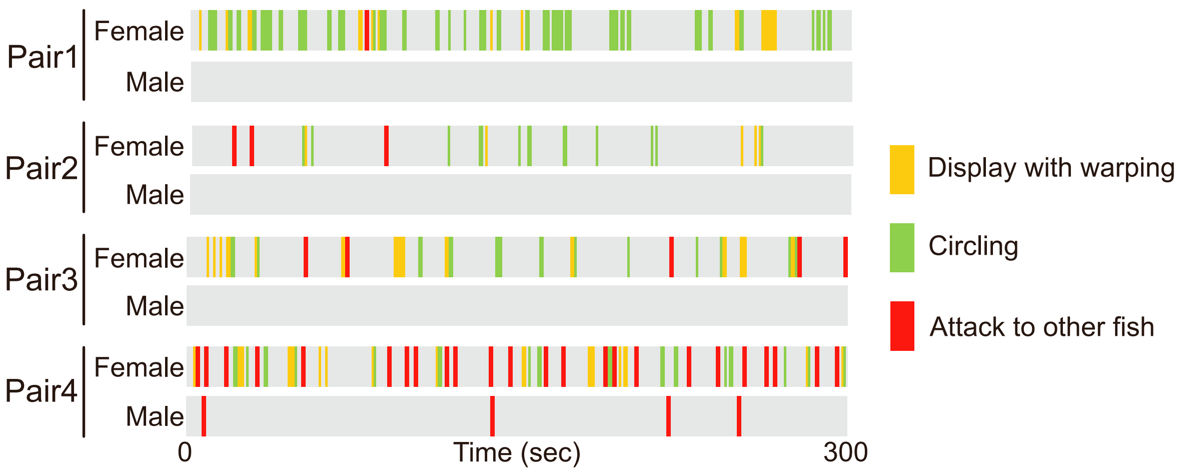

Figure S10 Time-course data of 5-min analyses of sexual behavior are shown as raster plots, related to discussion and Figure 1.

The yellow, green, and red bands indicate the timing and duration of display with warping, circling and attack to other fish, respectively.

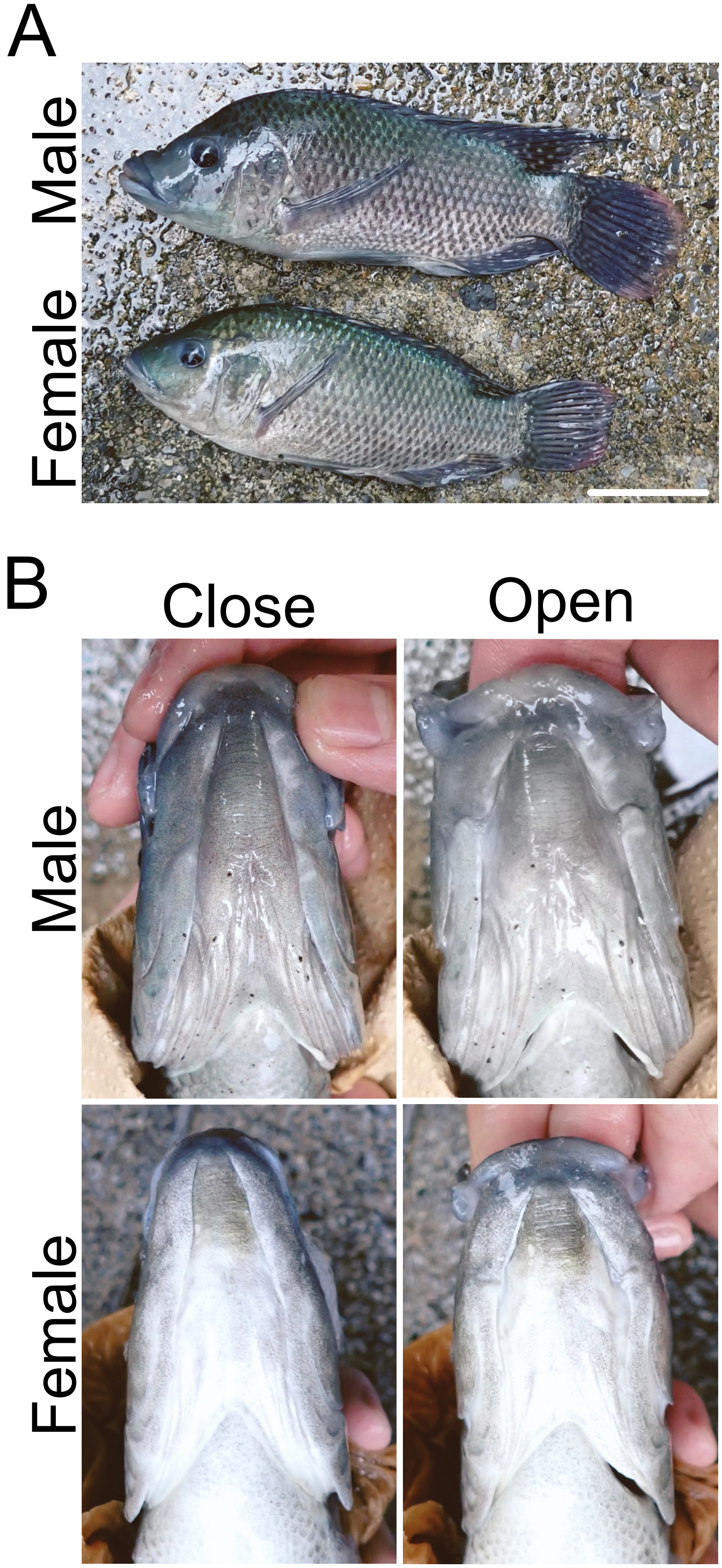

Figure S11 Another mouthbrooder, Mozambique tilapia (*Oreochromis mossambicus*; maternal mouthbrooder), does not exhibit sex-specific changes in the transparency of the lower jaw, related to discussion and Figure 1.

(A) Whole-body photograph of mature Mozambique tilapia. (B) The lower jaws of male and female Mozambique tilapia, which exhibit typically thicker and more opaque lower jaws.

Table S3 List of primer sequences, related to Figures 2 and 4.

Primer used for RT-qPCR

Name	Sequence (5' to 3')
actb_F	AGCACAGTGTTGGCGTACAG
actb_R	CCTTCCTTCCTCGGTATGGA
pnp4a_F	CTGGTATTCGGAGAGTTGAAGG
pnp4a_R	AACTGGGAACGTTGTCTTACAGAG
ara_F	GCTCTATGCTCTACTTTGCTCCA
ara_R	ACCTTCAGCATACAAAACCTCTG
arb_F	GAAGAACTCCATGGGGAACA
arb_R	GAAGGCAGAGGTGGGAGAGT
alkal2a_F	AAAGATGTGCACGGCTTCTC
alkal2a_R	GACAACTCGTATTTGGCTTCAAC

Primer used for RTPCR

Name	Sequence (5' to 3')
ara_F	GAGTCTGATCCGTTGGATAC
ara_R	ACAGGTGGTTCTGCTTACCT
arb_F	ACCGTGTCCTGCTCTTATGG
arb_R	TGAAACCTGGGAGTCCTTTG
eef1a1_F	GCCTACATTAAGAAGATCGGTTACA
eef1a1_R	TCTTCTCCACTGACTTGATAACACC

Primer used for cloning for in situ hybridization

Name	Sequence (5' to 3')
pnp4a_F	GCTGATTGGCTGATGTCTCA
pnp4a_R	GGCAAAACCAATCAACATGA
alkal2a_F	GCTTCCTGTAACCGCGTATC
alkal2a_R	TGTGTGCTACCCTTCTGTGC
ara_F	GAGTCTGATCCGTTGGATAC
ara_R	ACAGGTGGTTCTGCTTACCT
